# Supplementary figures and images for: Using prototyping to choose a bioinformatics workflow management system
Source: PLoS Comput Biol. 2021 Feb 25;17(2):e1008622. doi: 10.1371/journal.pcbi.1008622 (PMC7906312; doi:10.1371/journal.pcbi.1008622)

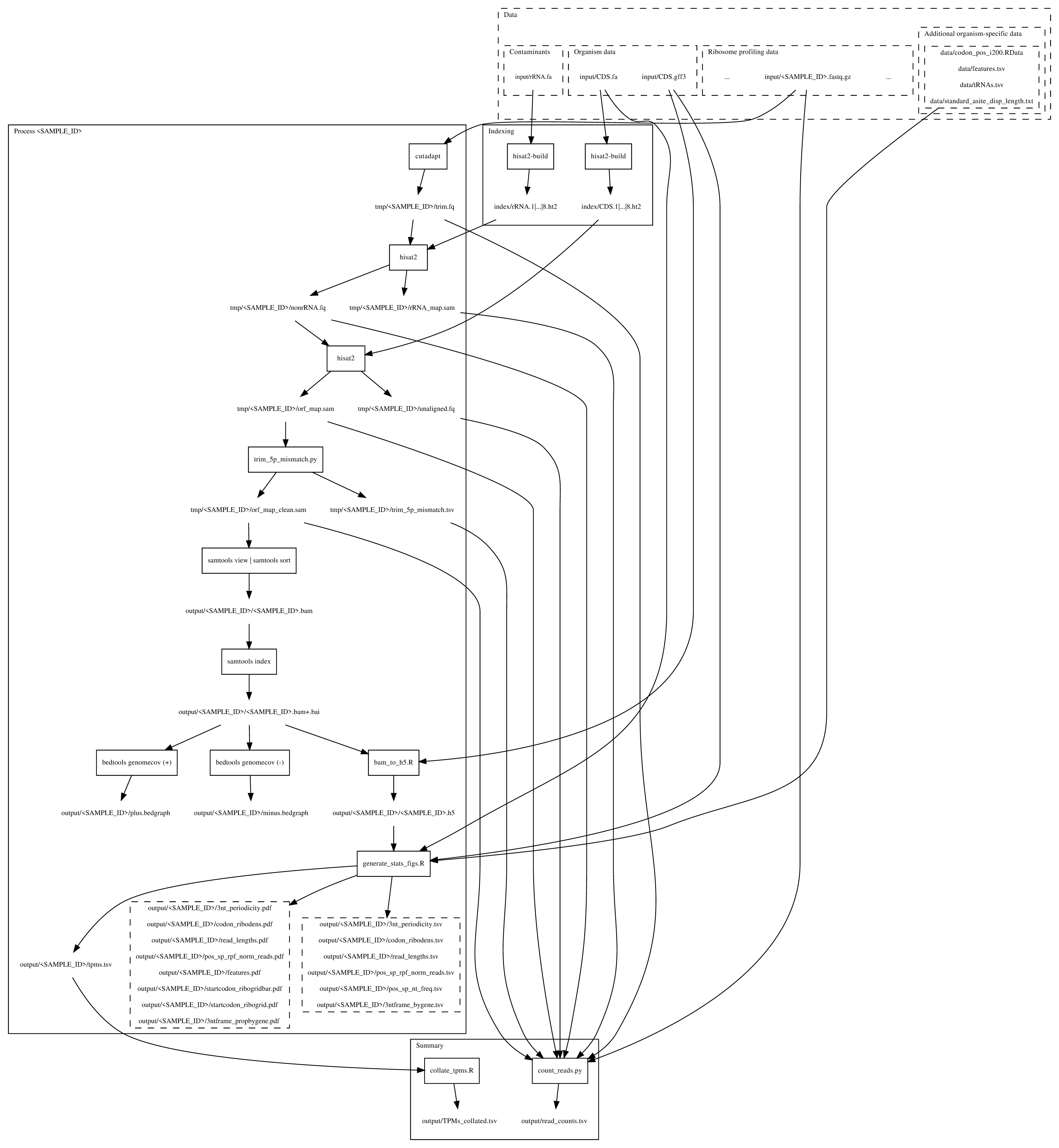

Supplement: S1 Fig — (TIF) [file pcbi.1008622.s001.tif]

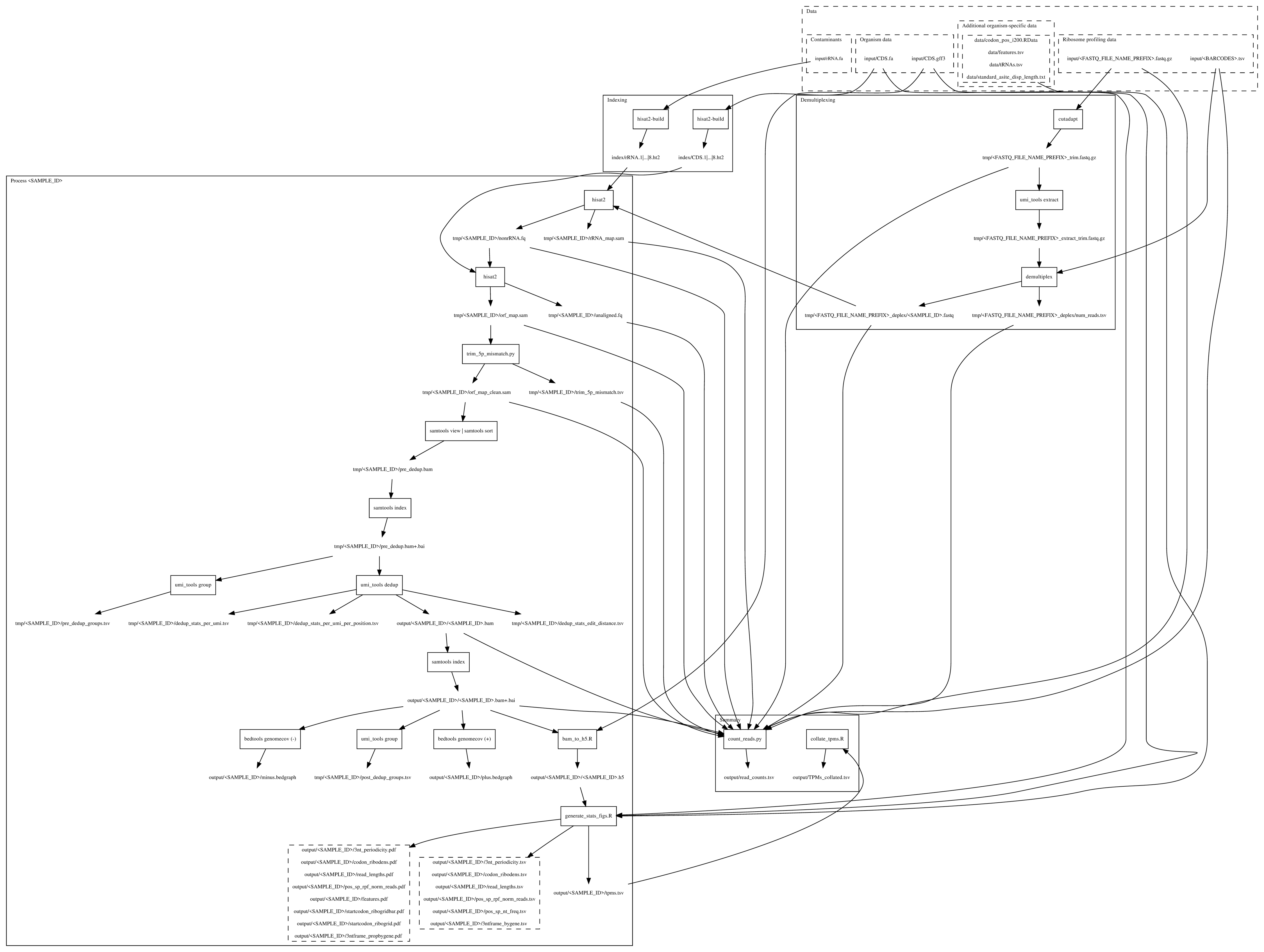

Supplement: S2 Fig — (TIF) [file pcbi.1008622.s002.tif]
